# Waterproof Fabric‐Based Multifunctional Triboelectric Nanogenerator for Universally Harvesting Energy from Raindrops, Wind, and Human Motions and as Self‐Powered Sensors

**DOI:** 10.1002/advs.201801883

**Published:** 2019-01-04

**Authors:** Ying‐Chih Lai, Yung‐Chi Hsiao, Hsing‐Mei Wu, Zhong Lin Wang

**Affiliations:** ^1^ Department of Materials Science and Engineering National Chung Hsing University Taichung 40227 Taiwan; ^2^ Innovation and Development Center of Sustainable Agriculture Research Center for Sustainable Energy and Nanotechnology National Chung Hsing University Taichung 40227 Taiwan; ^3^ School of Materials Science and Engineering Georgia Institute of Technology Atlanta GA 30332 USA; ^4^ Beijing Institute of Nanoenergy and Nanosystems Chinese Academy of Sciences National Center for Nanoscience and Technology (NCNST) Beijing 100083 P. R. China

**Keywords:** raindrop energy, smart clothes, triboelectric nanogenerators, wearable energy, wind energy

## Abstract

Developing nimble, shape‐adaptable, conformable, and widely implementable energy harvesters with the capability to scavenge multiple renewable and ambient energy sources is highly demanded for distributed, remote, and wearable energy uses to meet the needs of internet of things. Here, the first single waterproof and fabric‐based multifunctional triboelectric nanogenerator (WPF‐MTENG) is presented, which can produce electricity from both natural tiny impacts (rain and wind) and body movements, and can not only serve as a flexible, adaptive, wearable, and universal energy collector but also act as a self‐powered, active, fabric‐based sensor. The working principle comes from a conjunction of contact triboelectrification and electrostatic induction during contact/separation of internal soft fabrics. The structural/material designs of the WPF‐MTENG are systematically studied to optimize its performance, and its outputs under different conditions of rain, wind, and various body movements are comprehensively investigated. Its applicability is practically demonstrated in various objects and working situations to gather ambient energy. Lastly, a WPF‐MTENG‐based keypad as self‐powered human–system interfaces is demonstrated on a garment for remotely controlling a music‐player system. This multifunctional WPF‐MTENG, which is as flexible as clothes, not only presents a promising step toward democratic collections of alternative energy but also provides a new vision for wearable technologies.

Harvesting renewable and clean energy resources in natural is a challenge and urgent need for relieving the consequences from fossil energy.^1^, ^2^, ^3^, ^4^ In addition, for living and applications in remote locations, harvesting locally accessible energy is more favorable than constructing power plants that requires significant investments and infrastructure.^5^, ^6^, ^7^, ^8^, ^9^, ^10^, ^11^ Solar energy can be the natural option for the places where reach plenty of sunshine.^12^, ^13^, ^14^, ^15^, ^16^ However, lots of places on earth locate with either high precipitation or seasonally varied sunshine length. For example, for places with maritime or tropical climate as Western Europe or South American, yearly rain rates can exceed 2 m and precipitations disperse throughout the year.^5^ For places at high and middle latitudes as Berlin and Beijing, the differences of daylight time between summer and winter can exceed 5 h. In such cases, other natural energy such as rains and winds can be alternative resources.^6^, ^7^, ^8^, ^9^, ^10^ However, traditional hydropower plants require proper terrain and huge investments to build dams and barrages, also damaging natural environment. Wind turbines are limited by large volume, high costs, and inscrutable wind direction. Moreover, conventional generators are designed based on heavy, stiff, and inflexible materials, blocking their deployment on rugged landforms, buildings, nonplanar, and universal surfaces.^15^, ^16^, ^17^, ^18^, ^19^, ^20^, ^21^ Toward democratic and decentralized energy, it is highly desired to develop local energy harvesters that are nimble, downscaling, shape adaptable, widely implementable, cost‐friendly, effective, and able to universally gather different alternative resources.^4^, ^5^, ^6^, ^7^, ^8^, ^9^, ^10^, ^12^, ^22^

On the other hand, personal smart devices, such as wearable/mobile/biomedical sensors and electronics, are rapidly developing.^12^, ^23^, ^24^, ^25^ Those electronics are typically powered by rechargeable batteries. Yet, conventional batteries suffer from not only heavy weight and bulky volume but also limited capacity and lifetime as well as toxic wastes, impeding the progress and sustainable uses of next‐generation devices. Ideally, the need of electric energy can be all or at least partially gained from ambient energy such as human motions or others.^3^, ^12^, ^13^, ^14^, ^25^, ^26^

Triboelectric nanogenerators (TENGs), which can convert mechanical energy into electricity based on triboelectrification and electrostatic induction, have been considered as effective technology to harvest ambient energy and realize self‐powered sensors.^6^, ^7^, ^8^, ^27^, ^28^, ^29^ Their superiority includes wide material availability, excellent reliability, low cost, and flexible usage. Particularly, they show remarkable capability in effectively collecting irregular or low‐frequency (<5 Hz) mechanical energy, acting as new green energy providers.^30^ Developing energy device based on fabrics can endow them with fabrics' advantages including flexibility, shape adaptability, deformability, wearability, and lightweight, enabling them to accommodate intricate deformations from nonconventional substrates or human motions, and enlarge their applicability.^3^, ^12^, ^13^, ^14^, ^25^, ^26^ Not only wearable energy,^25^, ^26^, ^31^, ^32^, ^33^, ^34^, ^35^ but also recent efforts have started to focus on developing fabric‐based TENGs (f‐TENGs) for natural energy.^9^, ^36^ For example, Xiong et al. reported a single‐electrode‐mode f‐TENG for collecting energy from flowing water.^36^ However, because water molecules adhered onto the device surface can stop triboelectric effect and fatally annihilate the capabilities of TENGs, the fabric required a troublesome hydrophobicity process.^37^ Even so, hydrophobicity becomes ineffective after long‐term uses and washings. Due to the same issues, although a flag‐type TENG was developed to extract wind energy from various directions, its capability seriously degraded in humid and rainy weather.^9^ Such water‐caused malfunction can also easily occur on wearable f‐TENGs because of sweat or external weather.^26^, ^31^, ^32^Besides, previous TENGs/f‐TENGs can only harvest energy from one designated source, largely limiting their potentials and uses.^9^, ^25^, ^26^, ^31^, ^32^, ^33^, ^34^, ^35^ Recent significant progress reported that the combination of fabric‐based photovoltaics and f‐TENGs brought a new type of power textile that can simultaneously harvest energy from sunlight and human motions;^12^, ^13^, ^14^ however, the process for integrating two different devices raises difficulties in industrial manufacturing, also its potential is limited by the issues of solar energy. A “unitary” fabric‐based energy harvester that can extract energy from both natural, like rains and winds, and mechanical motions can be an important step for democratic and decentralized energy and wearable technologies due to the flexibility and compliance in both forms and uses; however, until now, there is no such example. Further, toward practical level, key issues of f‐TENGs, including waterproof, manufacturability, and reliability and stability for various weathers, require to be addressed.

Herein, we present a waterproof and fabric‐based multifunctional TENG (WPF‐MTENG) that can harvest energy from rains, winds, and various human movements and use as wearable self‐powered interfaces. To the best of our knowledge, the WPF‐MTENG is the first “single” TENG that possesses the advantages of waterproof and fabric characteristics and enable us to scavenge energy from both natural tiny impacts (rains and winds) and human motions. The combined capabilities and merits enable us to break the limitations from forms, water, and weathers to collect various energy resources, largely broadening the using spectrum of energy devices in either alternative or wearable energy uses. The WPF‐MTENG is comprised of multilayered fabrics and works at a contact‐separation mode.^6^, ^38^, ^39^, ^40^ The fabrics' soft and elastic features empower to turn gentle impacts from raindrops, winds, and human motions into contact separation of two internal active fabrics and result in electric outputs. A designing strategy for optimizing contact‐separation‐mode f‐TENG is proposed. The outputs at different conditions of rainfalls, winds, and body motions are comprehensively investigated. Producing electric powers and energy from different sources is evaluated and stored in capacitors. Further, the applicability is explored in diverse objects, including an umbrella, a raincoat, a building's exterior, a flag, a sole, and so forth, to practically harvest different energy resources, revealing their very wide application prospects. Last, to present its potential in active fabric human interfaces, a wearable WPF‐MTENG‐based keypad is demonstrated on a garment for wirelessly controlling a music‐player system. The multifunctional yet nimble WPF‐MTENG can not only address the long‐lasting challenge of waterproof, adaptive, deformable, and universal energy devices for locally accessible energy, but also bring a new class for wearable energy and smart fabric articles.

All of the materials and process are feasible for manufacturing. **Figure** **1**a schematically illustrates the fabrication process and its applications in harvesting rain, wind, and human‐motion energy. Detail is in the “Experimental Section” in the Supporting Information. Figure 1b shows an exploded view of the multilayered WPF‐MTENG, which is mainly constructed of two laminated fabrics. A laminated fabric means a combined fabric that is composed of one fabric and one or more continuous polymeric layers on its surfaces.^41^ The bottom combined fabric consisted of a conducting fabric sandwiched between an ethylene–vinyl acetate (EVA) film as underlay and a roughened rubber membrane. Figure 1c shows a side‐view scanning electron microscope (SEM) image of the bottom laminated fabric. The top laminated fabric was composed of a conducting fabric sandwiched between a mesh fabric and an EVA film. The WPF‐MTENG was assembled by placing the mesh side of the top part onto the bottom rubber membrane and gluing the borders of the two combined fabrics by a waterproof adhesive. Different from previous f‐TENGs, in which the conducting fabrics were fabricated by physically deposited metals or solution‐coated conducting nanomaterials,^3^, ^9^, ^12^, ^13^, ^14^, ^25^, ^36^ the conducting fabrics used here were obtained by coweaving silver fibers and lyocell rayon. The inherent conducting feature ensures the reliability and durability of WPF‐MTENGs while they were deformed for various uses. Figure 1d shows a SEM image of the conducting fabrics; the bright wires are silver fibers (diameters, ≈90 µm). Figure 1e shows the top‐view SEM image of the EVA film. The EVA surface is a continuous porous surface yet no penetration hole, enabling its waterproof property and acting as the encapsulation layer. The micrometer porous surface on the EVA film is designed for stopping the penetration of water drops and avoiding the adherence on skin when it is worn on human body.^42^ The bottom roughened rubber membrane is utilized as the triboelectrically charged layer. The roughened surface was obtained by molding via SiC papers. Figure 1f shows a top‐view SEM image of the roughened rubber membrane that was molded by a 425 µm SiC paper. Figure S1a–f (Supporting Information) shows more detail about the SEM images of SiC papers with different SiC grit sizes and the corresponding molded rubber membranes. Our previous study has shown that rubber is a superior triboelectrically negative material and possesses excellent soft and elastic features so that it can retain the flexibility and compliance of fabrics.^29^, ^32^ These characteristics are important for conformal deployment of nonconventional surfaces. Note that the rubber membrane was molded by separated SiC papers, and intervals between the molded areas were constructed rubber spacers. The SiC grit sizes on the molding papers and the areas of patterned units as well as the heights of spacers were investigated to optimize the performance. The mesh fabric was used for ensuring the effective separation of the two parts and maximizing the output electricity. Figure S2 (Supporting Information) shows an image of the mesh fabric. Figure 1g,h shows the resulting WPT‐MTENG and the demonstrations of its flexibility, including being rolled up, twisted, crumpled, and stretched.

**Figure 1:**
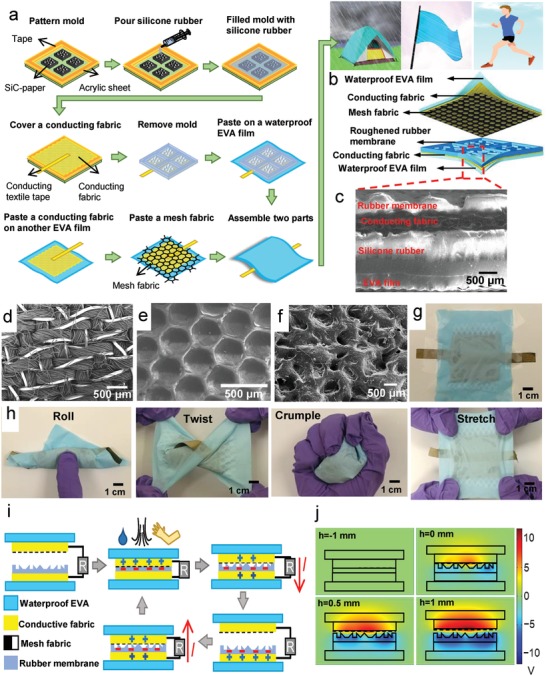
a) Schematic fabrication process and illustration of its applications. b) Exploded view of WPF‐MTENG. c) Cross‐view SEM image of bottom part of WPF‐MTENG. d) SEM image of conducting fabric. Bright wires are silver fibers with a diameter of ≈90 µm. e) SEM image of EVA film. f) SEM image of roughened rubber membrane. g) Photos of as‐prepared WPF‐MTENG. h) Demonstrations of flexibility, including being rolled, folded, twisted, crumpled, and stretched. i) Schematic illustration of working mechanism. j) Simulation results of electric potential difference between two active layers during contact and separation at open‐circuit condition.

Figure 1i schematically depicts its working mechanism which involves contact triboelectrification and electrostatic induction.^6^, ^7^, ^8^ Different from the previous single‐electrode‐mode f‐TENG for harvesting flowing‐water energy,^15^, ^16^, ^36^ the WPF‐MTENG is operated at contact‐separation mode in which the top conducting fabric and bottom rubber membrane are acted as two active triboelectrification materials. A deep study about the mechanism of the contact‐separation‐mode TENG can be found in the previous reports.^6^, ^38^, ^39^ Initially, before two active materials were in contact, there was no charge transferred. When external impacts that come from raindrops, winds, or human motions were applied to WPF‐MTENG, the air in fabric interspaces shifted, and the top conducting fabric came contact with bottom rubber membrane. At this stage, electrons transferred from the top conducting fabric to the rubber surface because of the highest electron affinity of rubber,^7^ resulting in the positively triboelectric‐charged conducting fabric and negatively triboelectric‐charged rubber. The two materials remained in electrostatic equilibrium state, and no potential difference was formed as they were in contact. After relieving external impacts, the air moved back to its initial interstitial spaces and separated the top conducting fabric and rubber, leading to an electrical potential difference between two active materials. Because the top positively triboelectric‐charged conducting fabric has a higher potential than the bottom one, electrons started to flow from the bottom to top conducting fabric through external circuit, leading to a current flow to the bottom electrode. As contact occurred again, current flowed reversely. Successive contact and separation of the two triboelectrification materials through external impacts can continuously produce electric outputs. Figure 1j shows the finite‐element simulation of the working mechanism at open‐circuit condition. Detailed simulation method is discussed in the Supporting Information. It can be found that there is a change of electric potential difference between the top and bottom conducting textiles during contact and separation of the two parts. The change of electric potential difference can also induce an electricity output.

**Figure** **2** shows the outputs from harvesting rainfall energy. Raindrops typically carry two types of energy: i) the kinetic energy as they fall and ii) the electrostatic energy generated during electrification with air.^5^, ^10^ The WPF‐MTENG was designed for gathering the former one. Competing previous single‐electrode‐mode TENGs/f‐TENG for collecting the latter one suffers in term of unreliability of the active layers' hydrophobicity.^15^, ^16^, ^36^ The contact‐separation‐mode WPF‐MTENG encapsulated in the waterproof EVA films did not need tortuous hydrophobic process, not only simplifying the process but also ensuring its reliability and durability. The soft and elastic fabrics enable us to extract the tiny impact energy from raindrops. Figure 2a illustrates the configuration for collecting rain energy. Output open‐circuit voltage (*V*_oc_) and short‐circuit current (*I*_sc_) were measured by the unit area of WPF‐MTENG. First, WPF‐MTENGs were optimized through patterning the rubber membranes with different SiC grit sizes and using the mesh fabric. For a standard test, water drops sprayed from a sprinkler were used to simulate the rainfall and set at 68.4 mL s^−1^ to the normal direction of WPF‐MTENG. As shown in Figure 2b and Figure S3 (Supporting Information), the outputs from the WPF‐MTENG constructed by a larger‐grit‐patterned rubber membrane and a mesh fabric exhibited the maximum performance. Here, the rubber surfaces were roughened for two functions: i) creating air gaps between the fabrics for the purpose of TENG and ii) increasing contact areas with the top conducting fabric.^39^ These two results were trade‐offs. The rubber membrane patterned by coarser SiCs possessed less contact areas, but, relatively, it created larger air gaps. It was found that the effective air gaps between the fabrics play a dominant factor to outputs. This is because rubber revealed very strong triboelectrically charged characteristic, although this feature can provide high performance for an f‐TENG device, the large amount of triboelectric charges on the rubber surface made the top conducting fabric tend to adhere onto the rubber, obstructing the two active layers from separation. The larger‐grit‐molded rubber membrane possessed rougher surface and formed bigger air gaps between the fabrics, enabling to effectively separate two active layers after they were in contact. And, the additional mesh fabric can form extra effective air spacers within the WPF‐MTENGs, further maximizing the outputs. The outputs depending on the areas of patterned units are optimized in Figure S4a–c (Supporting Information). The outputs depending on the heights of spacers are shown in Figure S5a–c (Supporting Information). These results also suggest that the design of effective and appropriate separating gaps between two active fabrics plays a key factor to the outputs of contact‐separation‐mode f‐TENGs.

**Figure 2:**
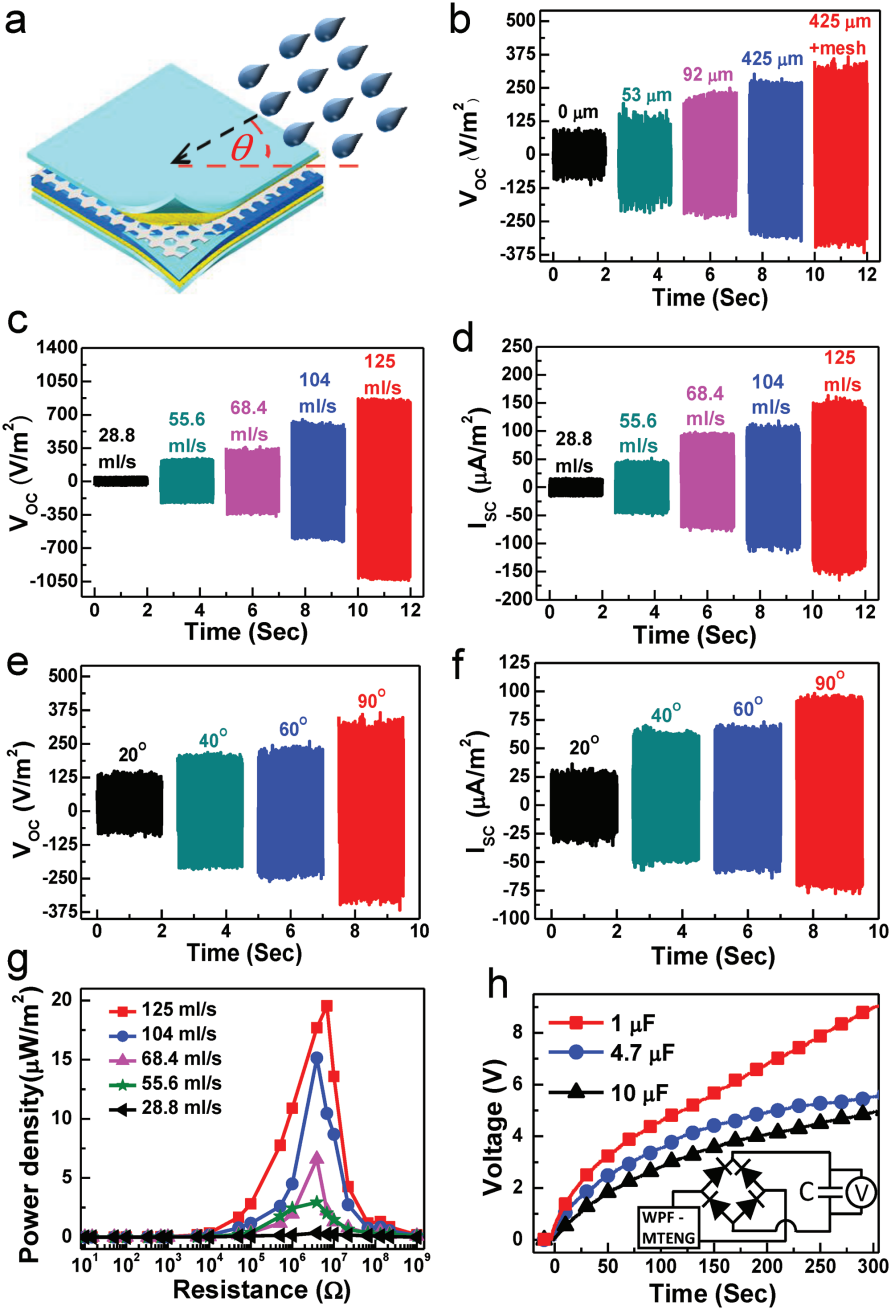
a) Schematic illustration for harvesting rainfall energy. b) *V*_oc_ of WPF‐MTENGs constructed by rubber membranes molded by different SiC‐grit sizes and with/without a mesh fabric. c) *V*_oc_ and d) *I*_sc_ at different rates of rainfalls. e) *V*_oc_ and f) *I*_sc_ at different angles of rainfall (68.4 mL s^−1^). g) Output power density at different external loads. h) Charging curves to different capacitors (rainfall, 125 mL s^−1^ from normal direction).

Figure 2c and Figure 2d show *V*_oc_ and *I*_sc_ of the optimized WPF‐MTENG at different rainfall conditions, respectively. As rainfall increased from 28.8 to 125 mL s^−1^, generating *V*_oc_ increased from ≈40 to ≈850 V m^−2^ and *I*_sc_ was obtained from ≈15 to ≈150 µA m^−2^. The outputs converted from single water drops (120 µL) can be found in Figure S6a,b (Supporting Information). Producing *V*_oc_ and *I*_sc_ at different rainfall angles is presented in Figure 2e and Figure 2f, respectively. The rainfall angle was measured from the plane of WPF‐MTENG, and the rainfall was set at 68.4 mL s^−1^. The results suggest that the WPF‐MTENG can generate more outputs when the relationship between the WPF‐MTENG and rainfall direction is more perpendicular. It is because the rainfall from the normal direction can contribute larger impacting forces, resulting in more efficient contact and separation between two active layers.

The generating electric power was explored by externally connecting different loads from 10 Ω to 1 GΩ in series and tested at different vertical rainfall (Figure 2g). The power was calculated as *I* ^2^ *R* (where *I* is the current across the load and *R* is the load resistance) and the producing current to different external loads are shown in Figure S7 (Supporting Information). As rainfall increases from 28.8 to 125 mL s^−1^, the maximum power increases from ≈0.35 to ≈19.53 µW m^−2^ and the optimum resistances from rain impacts locate in the regime of ≈3.9–6.8 MΩ.

The producing electricity can be stored in energy storage devices for future uses. Figure 2h shows the charging curve to different capacitors (at a rainfall of 125 mL s^−1^). The equivalent circuit for storing the producing electricity was in the inset of Figure 2h. The charged voltage reached up to ≈9 V in ≈300 s for a 1 µF capacitor, and, a 10 µF capacitor can be charged to ≈2 V in ≈60 s.

Figure S8 (Supporting Information) compares the outputs of the devices with and without laminating EVA films. The result indicates that the laminated waterproof EVA film plays an important role for the WPF‐MTENG to gather rain energy. Once water goes into the device, its capability to harvest energy will be annihilated.^37^ The durability of the WPF‐MTENG's waterproof property was further tested by two experiments: i) measuring its output after repeatedly washing the device and ii) measuring its output after immersing it in water for several days. For the test of washing the device, the challenge is the potential damage that may occur after continuously deformating and pulling the device during washing.^26^, ^32^ As shown in Figure S9a–c (Supporting Information), the outputs after five times washing show no significant decline, and the capability to harvest energy can be well retained. For the test of immersing the device in water for days, the challenge is that the potential penetration of water can destroy the energy‐harvesting ability of WPF‐MTENG. Figure S10a–c (Supporting Information) shows that its ability can be remained even after immersing into water for 5 days. These two results reveal the durability and reliability of its waterproof property.

Air flow that is wind can be the alternative and locally accessible energy resources. **Figure** **3** shows its performance from collecting wind energy. Figure 3a illustrates the configuration and wind direction measured. Figure 3b and Figure S11 (Supporting Information) show *V*_oc_ and *I*_sc_ from the WPF‐MTENGs with the rubber membranes that were patterned by different SiC grit sizes and with/without a mesh fabric, respectively. The tests were performed at a wind speed of 14.1 m s^−1^ from the normal directions of WPF‐MTENGs. Similar to the results in scavenging rain energy, the device constructed from a rougher rubber membrane and a mesh fabric can produce the optimal outputs from wind. Figure S12a,b (Supporting Information) shows the outputs to the spacers with different heights. The optimal WPF‐MTENG was then investigated its output *V*_oc_ and *I*_sc_ to different wind speed in Figure 3c and Figure 3d, respectively. As wind speed increased from 4.5 to 15.4 m s^−1^, *V*_oc_ increased from ≈90 to ≈900 V m^−2^ and *I*_sc_ increased from ≈50 to ≈150 µA m^−2^. Comparing to previously reported TENGs for wind energy, such as flutter‐driven,^17^ flag‐type,^9^ and lawn‐structured TENGs,^43^ the presented WPF‐MTENG can resist malfunction caused from humid and rainy weathers, which is the key property for practical applications.

**Figure 3:**
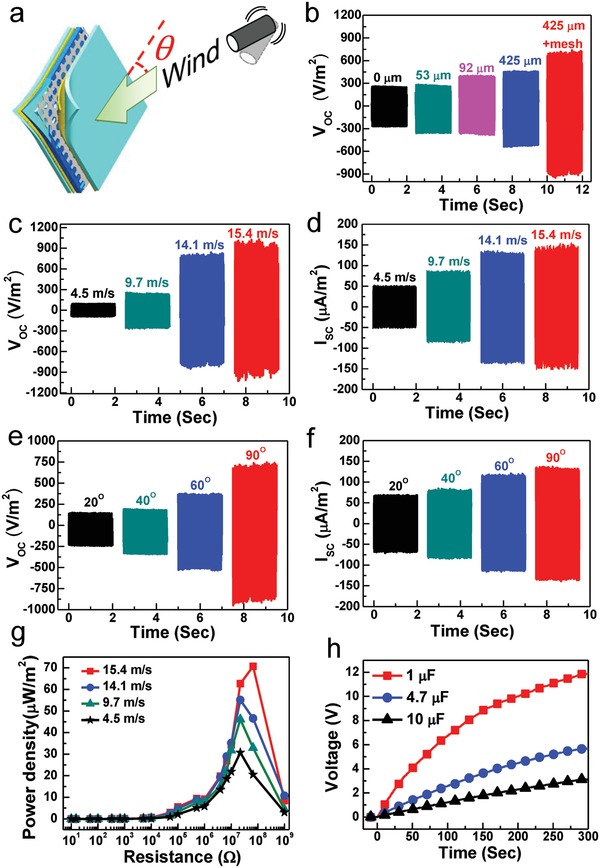
a) Schematic illustration for harvesting wind energy. b) *V*_oc_ of WPF‐MTENGs constructed by rubber membranes molded by different SiC‐grit sizes and with/without a mesh fabric. c) *V*_oc_ and d) *I*_sc_ at different wind speed. e) *V*_oc_ and f) *I*_sc_ at different angles of winds (14.1 m s^−1^). g) Output power density at different external load. h) Charging curves to different capacitors (wind speed, 15.4 mL s^−1^ from normal direction).

The laid WPF‐MTENG can collect wind energy from various directions. Its outputs from different‐direction winds (at 14.1 m s^−1^) are shown in Figure 3e,f. The larger wind force from the normal direction can induce more *V*_oc_ and *I*_sc_, which are consistent with the results in Figure 2e,f. The generating current and power to different loads are shown in Figure S13 (Supporting Information) and Figure 3g, respectively. As the wind speed increases from 4.5 to 15.4 m s^−1^, the maximum power increases from ≈30 to ≈70 µW m^−2^, and the optimum resistances from wind impacts are in the regime of ≈22–66 MΩ. It can be found that the optimum resistances from the outputs of winds are higher than the ones from rainfalls. Previous report indicated that the optimum resistance is inversely proportional to either the affected contact area or the average contact velocity.^39^ In the experiment, the area of the tested device was set at the same; therefore, it can infer that the average contact speeds caused from raindrops should be higher than the ones from air flow. The electric energy converted from wind can be stored, and the charging curves to different capacitors (at a wind speed of 15.4 m s^−1^) are shown in Figure 3h.

Not only can the flexible WPF‐MTENG be easily deployed for harvesting natural energy from rains and winds but also be readily introduced into clothing articles to collect energy from human motions, serving as waterproof wearable energy providers. The electricity produced from various human‐body movements was explored in **Figure** **4**. In the tests, a WPF‐MTENG with a size of 8 × 11.6 in.^2^ was worn on different parts of human body including the wrist, foot, elbow, and knee for scavenging various body‐movement energy. As shown in Figure 4a,b, tapping and stepping the worn WPF‐MTENG can produce *V*_oc_ to over 2000 V m^−2^ and *I*_sc_ to over 500 µA m^−2^. These results are comparable to with previous yarn‐based TENG (≈345 µA m^−2^) and fabric‐TENG (≈500 µA m^−2^).^34^, ^35^ When it was worn on the elbow and knee, it generated *V*_oc_ to ≈1100 V m^−2^ and *I*_sc_ to ≈ 25 µA m^−2^ during extending and flexing the arm and leg. The air permeability of the WPF‐MTENG is shown and discussed in Figure S14 (Supporting Information).

**Figure 4:**
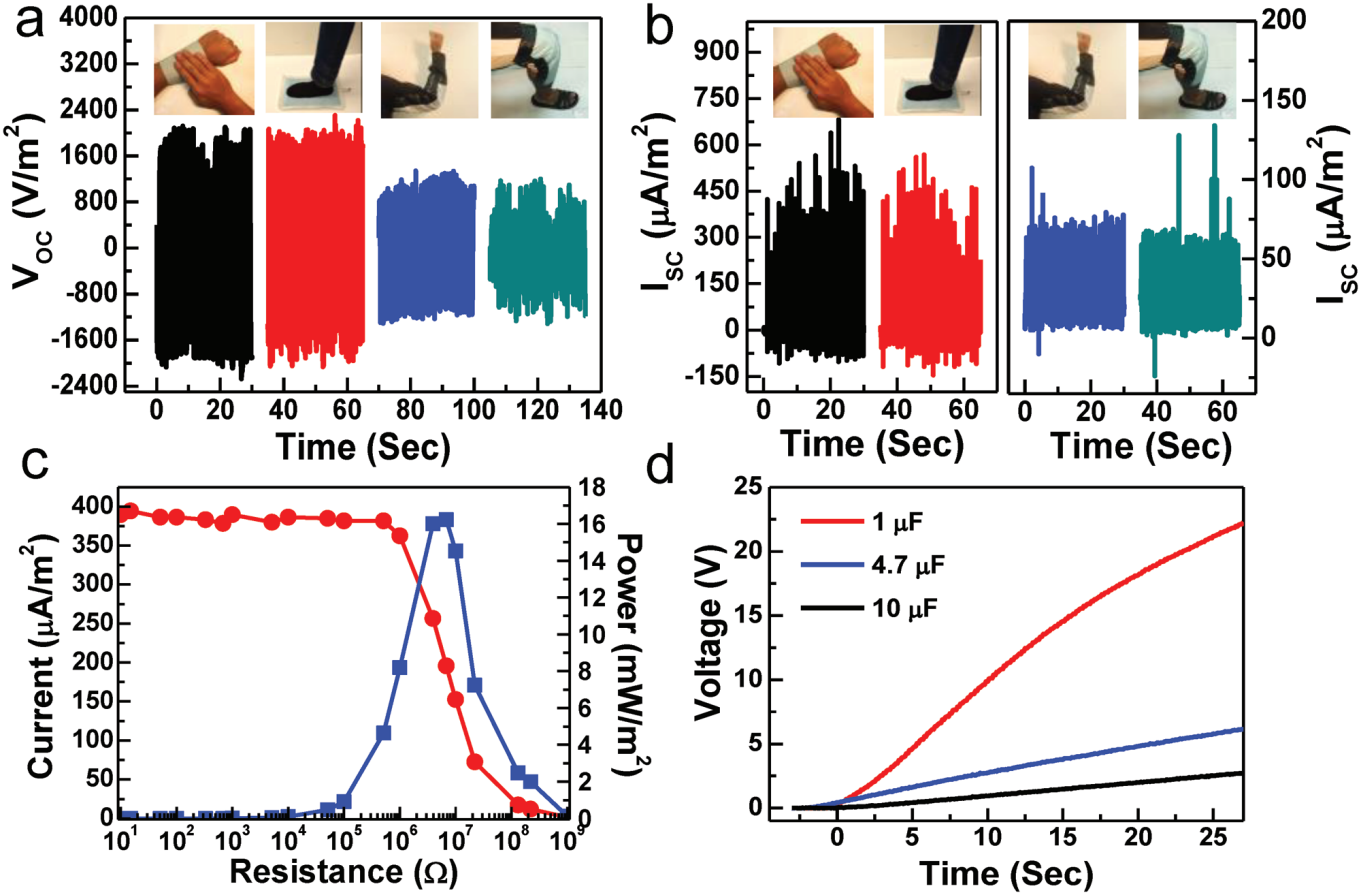
a) *V*_oc_ and b) *I*_sc_ from different body movements. c) Output current and power density at different external loads (tested by hand tapping). d) Charging curves to different capacitors (tested by tapping).

Figure 4c shows the generation of electric power to different loads through tapping. A maximum instantaneous power of ≈16 mW m^−2^ can be obtained at a 6.8 MΩ load. The electric energy harvested from tapping was demonstrated to be stored in different capacitors. As shown in Figure 4d, in 25 s, a voltage of over 20 V can be generated and stored in a 1 µC capacitor and a charged voltage of >5 V can be obtained for a 4.7 µC capacitor.

The durability of WPF‐MTENG was further examined through continuously loading and unloading a huge contact force of 30 N (≈3 kg) with a contact area of 5 × 5 cm^2^ (Figure S15, Supporting Information). No obvious degradation of outputs was found after repeatedly applying the huge impact forces over 1000 times, through which its durability can be confirmed.

The comparison of the outputs between WPF‐MTENG and the recently reported f‐TENGs is shown in Table S1 (Supporting Information). Although the outputs of the WPF‐MTENG are not higher than previous f‐TENGs that were designed for harvesting one designated energy source (rains/winds/human motions), it can gather energy from multisource and, particularly, against water‐caused malfunction, which can be more favorable for practical uses.

To demonstrate their applicability, WPF‐MTENGs were then practically exploited in different applications. First, to harness rainfall energy, the WPF‐MTENGs were applied in different fabric‐based rain gears including an umbrella (**Figure** **5**a) and a raincoat (Figure 5b). As shown in Figure 5a,b and Movies S1 and S2 (Supporting Information), the WPF‐MTENGs on the umbrella and raincoat can harvest water drops' impact energy and transform into electricity to light up tens of light‐emitting diodes (LEDs) for intuitively visible signals. These results suggest that WPF‐MTENGs can be used for designing self‐powered lighted rain gears that can protect human from traffic accidents caused by blurred vision in rainy days.

**Figure 5:**
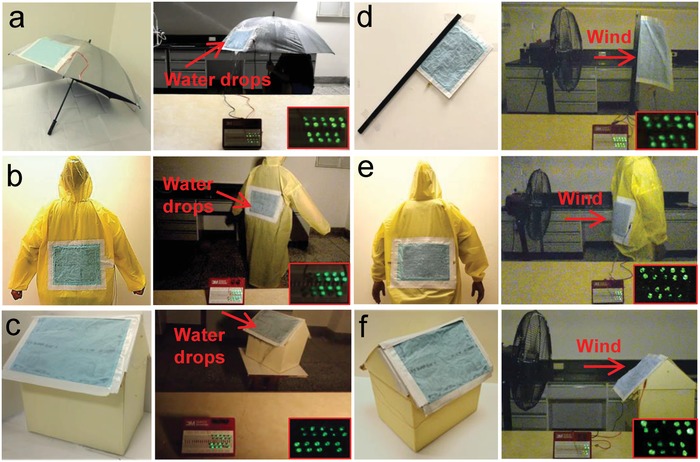
Demonstrations of WPF‐MTENGs in various practical uses: harvesting rain energy through a) an umbrella, b) a raincoat, and c) a roof; harvesting wind energy through d) a flag, e) a coat, and f) a roof.

The excellent mechanical compliance and flexibility from fabrics enable them to adaptively deploy on the appearance of buildings to collect rainfall energy. Figure 5c and Movie S3 (Supporting Information) demonstrate that the WPF‐MTENG deployed on the roof of a house model turned rainfall energy into electricity and lighted up tens of LEDs. Moreover, the foldable and rollable WPF‐MTENGs can be easily stored and spread out, enabling them to be nimbly used in the places where precipitations are seasonal, such as monsoon and plum rain.

To reveal the applicability in collecting wind energy, a flag fabricated by the WPF‐MTENG was demonstrated to harvest wind energy from a fan and produce electricity to power up LEDs (Figure 5d; Movie S4, Supporting Information). Comparing to wind turbines that require significant investments and wide plain to installation,^21^ the WPF‐MTENGs with extremely low energy cost show freedom in deployment and the capability to harvest wind energy from various directions.

Its unique waterproof property makes the WPF‐MTENG more suitable to harvest energy in humid and windy weathers. Figure 5e and Movie S5 (Supporting Information) demonstrate the WPF‐MTENG on a raincoat to extract wind energy. The results in Figure 5b,e clearly show the capability WPF‐MTENG on the coat to harvest energy from both rainfalls and winds. Based on our best knowledge, this is the first time that a wearable energy harvester can collect energy from both rains and winds.

Comparing with previously reported flag‐type and lawn‐structured TENGs that require to erect on the substrates to collect wind energy,^9^, ^43^ the WPF‐MTENG can be conformally deployed on building externals to collect wind energy. As shown in Figure 5f and Movie S6 (Supporting Information), the WPF‐MTENG adaptively placed on a house model's roof can convert wind energy to useful electricity. Above demonstrations illustrate that the WPF‐MTENGs can practically gather energy from both rains and winds, revealing their promising potential for decentralized energy uses.

The WPF‐MTENGs were then introduced into the sleeve of a coat and the insoles of shoes, respectively, to practically extract human‐motion energy. **Figure** **6**a and Movie S7 (Supporting Information) show the coat with a WPF‐MTENG generated electricity during flexion and extension of an elbow and power up LEDs. Figure 6b and Movie S8 (Supporting Information) demonstrate that the insoles fabricated by WPF‐MTENGs produced electricity and derived electronic components during stepping. These demonstrations clearly reveal their potential in wearable energy.

**Figure 6:**
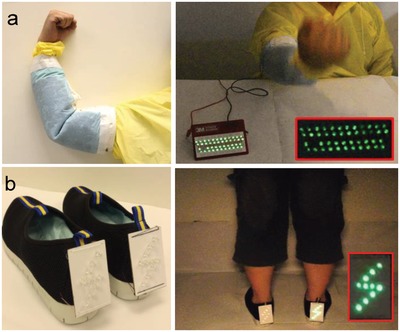
Demonstrations of WPF‐MTENGs in harvesting human‐motion energy through a) a garment and b) shoes.

Furthermore, touching a WPF‐MTENG can make the contact between two active fabrics and generate electrical signals. **Figure** **7**a,b shows the generating signals from a WPF‐MTENG (0.8 × 0.8 in.^2^) during finger touching and releasing. By progressing the actively responding signals, the WPF‐MTENG can serve as self‐powered human‐system interfaces. The fabric features enable us to readily integrate into a smart cloth. Figure 7c demonstrates a fabric‐based keypad composed of five WPF‐MTENGs (0.8 × 0.8 in.^2^ for each key) on a garment. The WPF‐MTENGs were also acted as music‐controlled keys, and integrated with a data‐processing microcontroller and a wireless transmitter for remotely controlling a music‐player system, as shown in Figure 7d. The circuit is shown in Figure S16 (Supporting Information). Pressing the “Play” key of WPF‐MTENG can remotely play the music in the player system (Figure 7e) and pressing the “Next” key of WPF‐MTENG can wirelessly play the next song of the system (Figure 7f). A comprehensive demonstration can be found in Movie S9 (Supporting Information). Competing conventional passive sensors for wearable uses suffers from lack of flexibility and wearability as well as the issues of the need of rigid batteries and power dissipation.^24^, ^44^ Competing previous f‐TENGs for wearable sensing suffers from troubles of water disturbance.^25^, ^26^ The presented WPF‐MTENG shows not only outstanding mechanical compliance and self‐powered vantages but also stability and reliability against water, which are key attributes for realizing next‐generation wearable technologies. Moreover, based on the fabric features, the WPF‐MTENGs are easily designed for diverse textile‐based articles, serving as smart textiles. Figure S17a and Movie S10 (Supporting Information) demonstrate a WPF‐MTENG‐based smart carpet that can be used for detecting the entering of people. Figure S17b and Movie S11 (Supporting Information) demonstrate a WPF‐MTENG‐based smart bedspread that can sense sleeper motions.^45^

**Figure 7:**
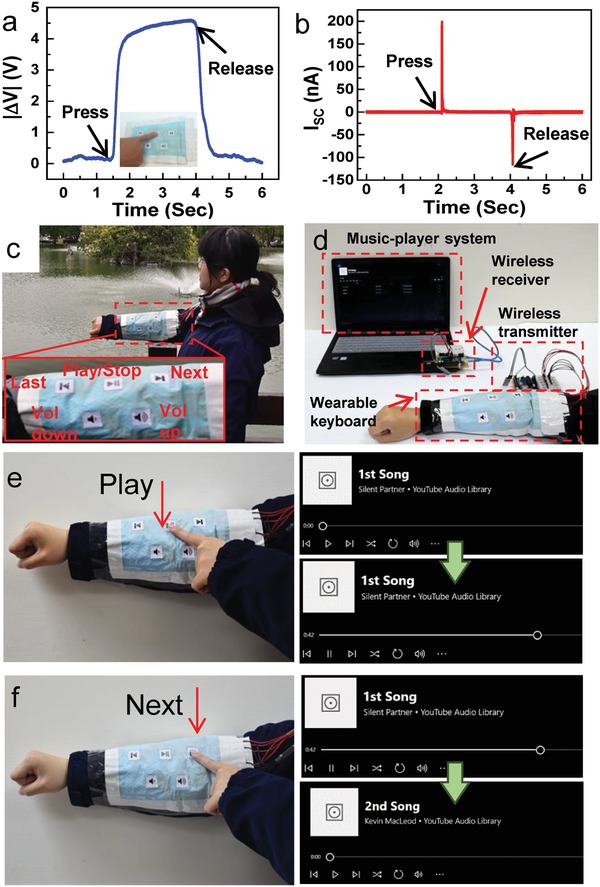
a) Output voltage and b) current during finger touching and releasing. c) Demonstrations of WPF‐MTENGs in use of fabric human–system interfaces. d) Photo of the wireless wearable WPF‐MTENG‐based music‐controlling keypad and music‐player system. e) Touching “Play” for wirelessly playing the first song. f) Touching “Next” for wirelessly playing the second song.

In summary, the first entirely fabric‐based and waterproof TENG that can simultaneously harvest energy from rains, winds, and various human movements has been demonstrated, which can not only be used as nimble, adaptive, deformable, wearable and universal energy collectors but also act as fabric self‐powered interfaces. Competing previous TENGs/f‐TENGs can only extract energy from one designated source and suffer from malfunction caused by water from sweat, humid, or rainy weather. The remarkable capability to harvest various energy resources together with the waterproof and fabric features enable us to collect ambient energy more freely, which can benefit the deployment of energy devices and boost their applicability in diverse applications. Its performance was optimized by constructing tiny air gaps between the fabrics. Their applicability was comprehensively demonstrated in various objects and working situations. Not only use as universal energy collectors, but also the WPF‐MTENGs were able to serve as fabric‐based active sensing interfaces for remotely communicating electronic systems. This work will not only enlighten the development of decentralized and remote energy but also promote a brand‐new wearable technology.

## Conflict of Interest

The authors declare no conflict of interest.

## Supporting information

Supplementary

Supplementary

Supplementary

Supplementary

Supplementary

Supplementary

Supplementary

Supplementary

Supplementary

Supplementary

Supplementary

Supplementary
