# Supplementary material for: Waterproof Fabric‐Based Multifunctional Triboelectric Nanogenerator for Universally Harvesting Energy from Raindrops, Wind, and Human Motions and as Self‐Powered Sensors
Source: Adv Sci (Weinh). 2019 Jan 4;6(5):1801883. doi: 10.1002/advs.201801883 (PMC6402409; doi:10.1002/advs.201801883)
Supplement: Supplementary file 1 — Supplementary [file ADVS-6-1801883-s002.pdf]

Copyright WILEY-VCH Verlag GmbH & Co. KGaA, 69469 Weinheim, Germany, 2019.

ADVANCED SCIENCE Open Access

Supporting Information

for *Adv. Sci.,* DOI: 10.1002/advs.201801883

Waterproof-Fabric-Based Multifunctional Triboelectric Nanogenerator for Universally Harvesting Energy from Raindrops, Wind, and Human Motions and as Self-Powered Sensors

Ying-Chih Lai, Yung-Chi Hsiao, Hsing-Mei Wu, and Zhong Lin Wang**

Copyright WILEY-VCH Verlag GmbH & Co. KGaA, 69469 Weinheim, Germany, 2016.

Supporting Information

Waterproof Fabric-based Multifunctional Triboelectric Nanogenerator for Universally Harvesting Energy from Rain Drops, Winds, and Human-Motions and as Self-Powered Sensors

Ying-Chih Lai,* Yung-Chi Hsiao, Hsing-Mei Wu, and Zhong Lin Wang*

Prof. Y.-C. Lai, Y.-C. Hsiao, H.-M. Wu,
Department of Materials Science and Engineering,
National Chung Hsing University
Taichung 40227, Taiwan
E-mail: yclai@nchu.edu.tw

Prof. Z. L. Wang,
School of Materials Science and Engineering
Georgia Institute of Technology
Atlanta, GA 30332, USA
E-mail: zhong.wang@mse.gatech.edu

Prof. Y.-C. Lai,
Innovation and Development Center of Sustainable Agriculture,
Research Center for Sustainable Energy and Nanotechnology,
National Chung Hsing University
Taichung 40227, Taiwan

Prof. Z. L. Wang,
Beijing Institute of Nanoenergy and Nanosystems
Chinese Academy of Sciences
National Center for Nanoscience and Technology (NCNST)
Beijing 100083, P. R. China

Experimental Section

Fabrication of waterproof TENG fabric: The conducting fabric was obtained by co-weaving silver fibers and lyocell rayon. To fabricate the triboelectrically charged material, parts A and B solutions of Eco-flex 30 silicone rubbers (Smooth-On, Inc.) were mixed in a ratio of 1:1 by weight. The liquid mixture of silicone rubber was poured onto an acrylic plate that was uniformly pre-attached with SiC-sandpapers as templates and bordered by 2-mm-thick foam tapes. The area of each attached sandpaper was set as 2×2 cm^2 and the interval between each sandpaper was set as 2 mm. The intervals (2-mm height; 2-mm width) here were used to form the rubber spacers for separating the top and bottom triboelectric contact materials. The mixture was then degassed for 10 minutes and covered with the as-prepared conducting fabric. After curing at room temperature for at least 4 hours, peeling off the conducting fabric from the acrylic plate, the roughened rubber was adhered on the surface of conducting fabric. Thereafter, the back side of the rubber-coated conducting fabric was glued with an ethylene-vinyl acetate (EVA)-based waterproof film (50-μm thickness, UNIDESIGN, Uni-President) by rubber solution. The resulting layered textile was acted as the bottom part of WPF-MTENG. On the other hand, another conducting fabric was adhered with a mesh fabric with a thickness of 200 μm. And, its back side was glued on another EVA film, acting as the top part of WPF-MTENG. Last, the mesh side of the top part was laid on the rubber side of the bottom part, and the WPF-MTENG was completed by assembling these two parts through gluing the borders of the top and bottom combined fabrics by waterproof adhesive (3M Scotch 6225N).

Characterization: The SEM images were characterized by Hitachi SU-8010. The open-circuit voltage was measured by a Keithley 6514 system electrometer, and the short-circuit current was measured by using an SR570 low-noise current amplifier (Stanford Research System). For the standard measurement in harvesting rain energy, the experiments were performed by sprinkling water drops at an altitude of 1 meter through a tunable showerhead and the WPF-TENG was tilted at 45 degree to horizon to avoid an accumulation of water film. For the standard measurement in harvesting wind energy, the WPF-TENG was fixed on an acrylic plate and applied wind force by shaking a hairdryer. And, the wind speed was measured by an anemometer (AVM-01, TES Electrical Electronic Corp.) For the demonstrations in fabric human-system interfaces, the WPF-TENGs were connected with a microcontroller (Arduino UNO) to process the actively responding signals.

Finite element simulation of the mechanism

The geometry for each simulation is drawn by the COMSOL software. A 2D model was used to simplify the simulation. When the silicone rubber membrane and the top conducting fabric are separated after they were in contact, the surface of rubber membrane and the top conducting fabric will have opposite charges due to the triboelectric effect. Based on the nature of triboelectric effect, the generated triboelectric charges are assumed to distribute uniformly on the rubber membrane, which is an excellent insulator. For the top conducting fabric, the internal resistance of the conducting fabric is neglected because the internal resistance of the conducting fabric is much smaller than the inherent impedance of the triboelectric nanogenerator.

Further, the decay of triboelectric charges with time can be neglected because the insulators can maintain the surface triboelectric charges for a long time. Due to the charge conservation, the surface of silicone rubber and the top conducting textile surface have the same amount of triboelectric charges. The whole structure is surrounded by air, which is the same as the case in the experiments. The potential at infinity is set as the reference point, which is 0, for the electric potential. The material property is also assigned in the COMSOL software. Subsequently, the required electrostatic properties can be calculated by utilizing the COMSOL software electrostatics module.

Parameters for the established models (these parameters will only affect the magnitude of the electric potential but not the changing trend of the calculated results): The thickness of the silicone rubber is set to be 2 mm. To present the roughened surface of the rubber membrane, the top surface of the rubber membrane is assumed to be a series of horizontal and oblique lines. The horizontal lines are individually set as 1 mm with the triboelectric charge densities of -80 nCm^{-2}. The oblique lines are individually set as 1.41mm with the triboelectric charge density of -59.2 nCm^{-2}. The length and thickness of the conducting fabric are set to be 16 mm $\times$ 2 mm, and the triboelectric charge density on the surface of conducting fabric is assigned to be 120 nCm^{-2}. The open-circuit voltage is derived by calculating the potential difference between the silicone rubber membrane and the top conducting fabric.

Supporting Information 1. Comparison of WPF-MTENG with recent fabric-TENGs

Triboelectric nanogenerators	Fabric based	Instantaneous power density from different sources (×: not allowed)			Ref
		Rain	Wind	Body motion	
Hydrophobic cellulose oleoyl ester nanoparticles - coated polyethylene terephthalate (PET) fabric	**O**	mW/m^2 ($6\ mLs^{-1}$)	×	×	[S1]
Water-drop TENG	×	mW/m^2 ($13mLs^{-1}$)	×	×	[S2]
Water-TENG	×	mW/cm^2 (flowing tap water)	×	×	[S3]
Freestanding flag-type woven TENG	**O**	×	mW/kg ($22\ m{\times}s^{-1}$)	×	[S4]
Flutter-driven TENG	×	×	mW/m^2 ($22\ m{\times}s^{-1}$)	×	[S5]
Lawn structured TENG	×	×	W/m^2 ($27\ m{\times}s^{-1}$)	×	[S6]
3D orthogonal woven TENG	**O**	×	×	mW/m^2 (~ 3 Hz)	[S7]
Single-thread-based TENG	**O**	×	×	mW/m^2 (~ 3Hz)	[S8]
TENG-cloth	**O**	×	×	mW/m^2 (~ 1 Hz)	[S9]
Yarn-based TENG	**O**	×	×	mW/m^2 (~ 3 Hz)	[S10]
Waterproof and fabric-based multifunctional TENG	**O**	$\mu W/m^2$ (125 ml/s)	$\mu W/m^2$ (15.4 m/s)	mW/m^2 (~ 1 Hz)	This work

Table S1 | Summary of recent fabric-based TENGs for different sources.

Supporting Information 2. SEM images of the SiC-papers and the patterned silicone rubber membranes

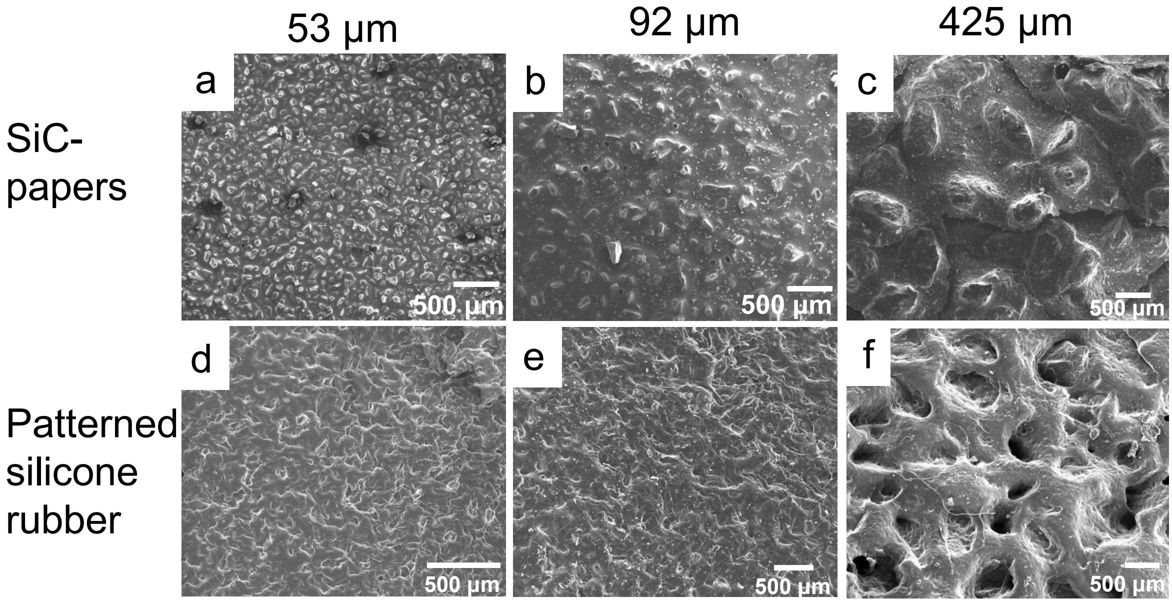

Figure S1. (a-c) SEM images of molding SiC-papers with different SiC-grit sizes. (d-f) Corresponding patterned rubber membranes.

Supporting Information 3. Images of the mesh fabric

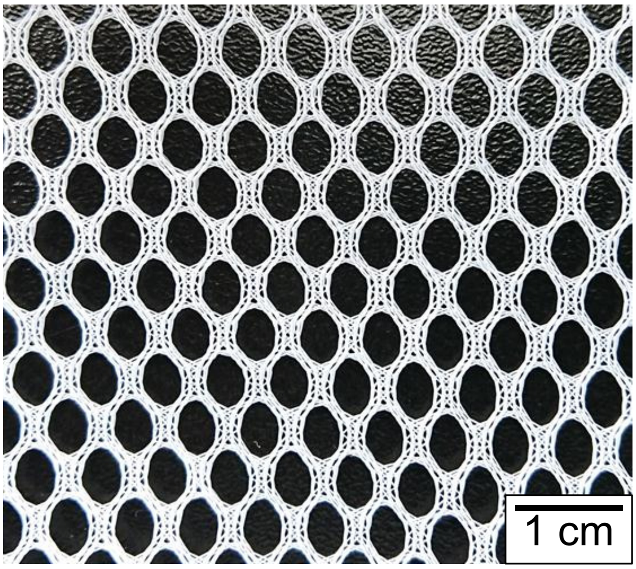

Figure S2. Photo of the sandwiched mesh fabric.

Supporting Information 4. I_{sc} of WPF-MTENGs at different structures

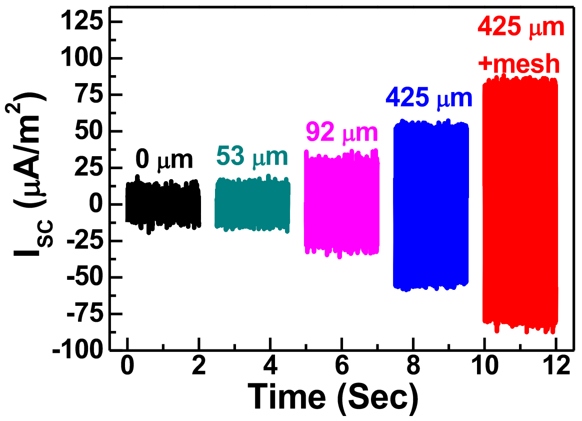

Figure S3. I_{sc} of WPF-MTENGs constructed by rubber membranes molded by different SiC-grit sizes and with/without an additional mesh fabric. Rainfall was set at 68.4 ml/s.

Supporting Information 5. Outputs of WPF-MTENG depending on the areas of patterned units

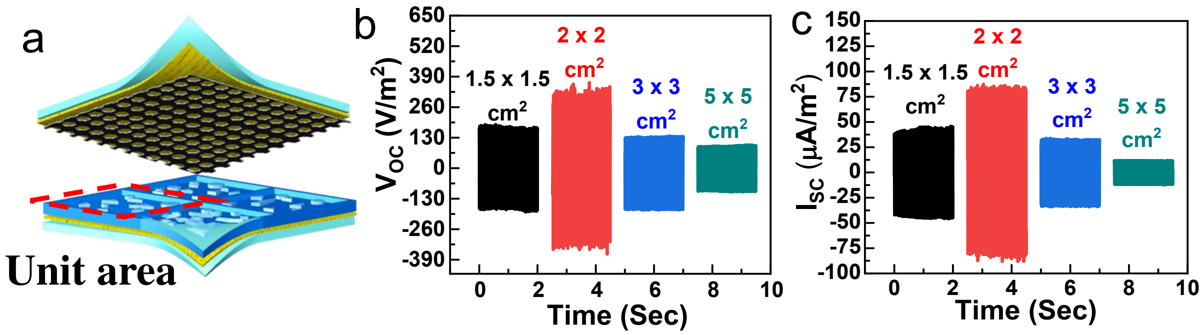

Figure S4. (a)Schematic illustration the patterned unit. (b) V_{oc} and (c) I_{sc} of WPF-MTENGs with different areas of patterned units.

Supporting Information 6. Outputs of WPF-MTENG depending on different heights of spacers

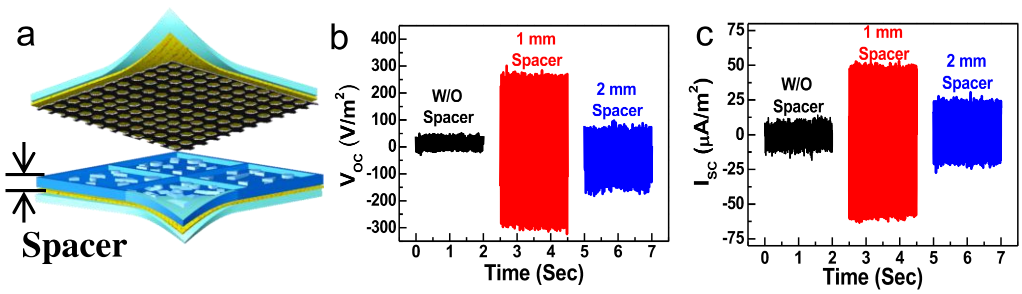

Figure S5. (a) Voc. (b) Isc of WPF-MTENGs with different heights of spacers at 68.4-ml/s rainfall.

Supporting Information 7. Outputs of WPF-MTENG from harvesting single water drop

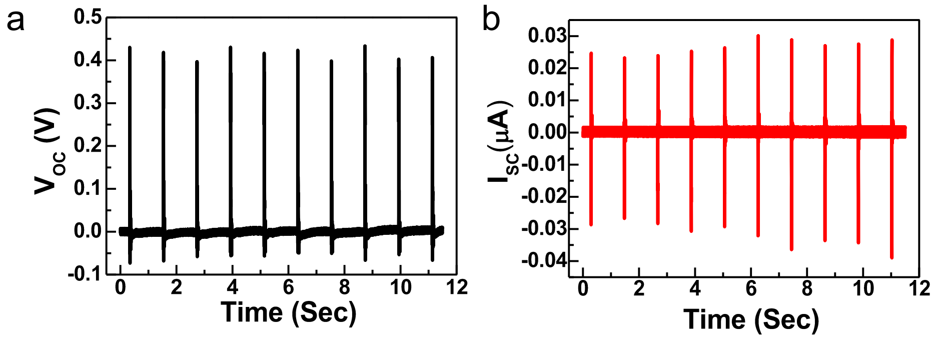

Figure S6. (a) V_{oc} , and (b) I_{sc} from harvesting single water drop (120 μl from a height of 40 cm, 1 Hz).

Supporting Information 8. Output current of WPF-MTENG to different external load

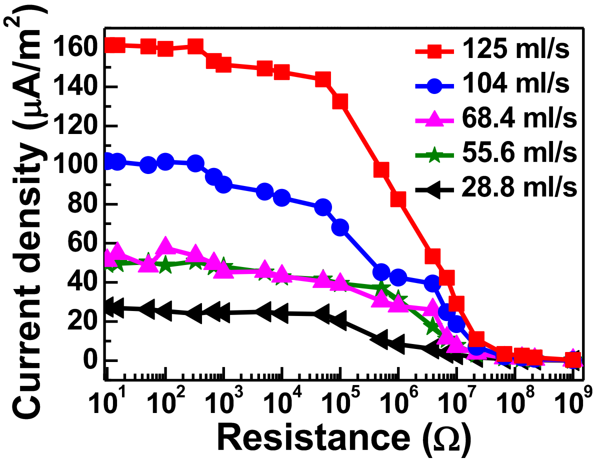

Figure S7. Output current of WPF-MTENG to different external load.

Supporting Information 9. Comparison of the outputs of the devices with and without laminating EVA films

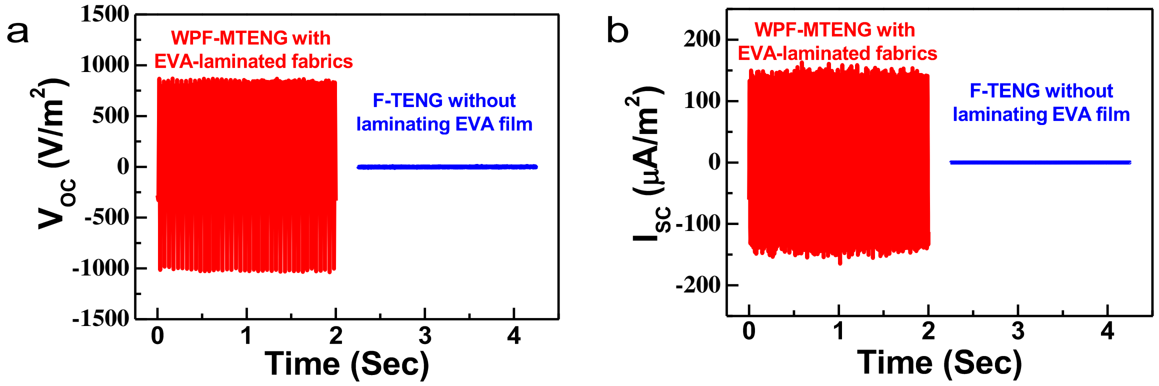

Figure S8. (a)V_{oc} and (b)I_{sc} from the devices with and without laminating EVA films (at rainfall of 125 ml/s).

Supporting Information 10. Outputs of WPF-MTENG after repeatedly washing

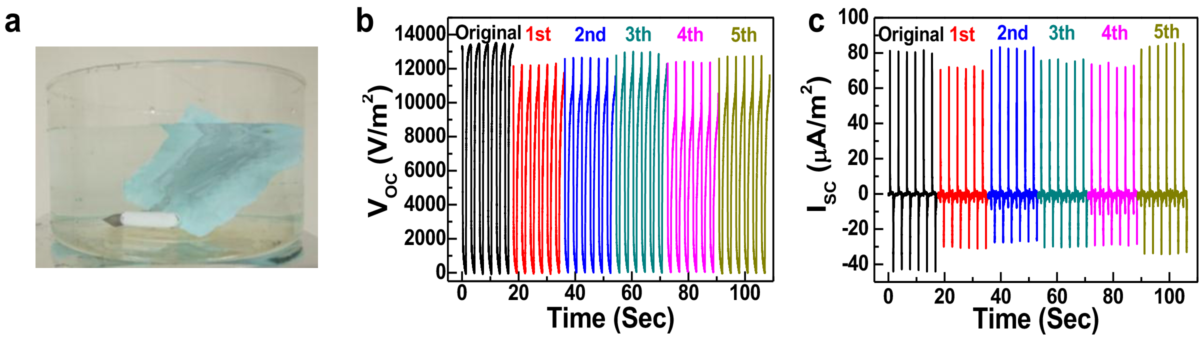

Figure S9. (a) Photo of washing the WPF-MTENG by stirring bar. (b)V_{oc} and (c)I_{sc} of WPF-MTENG after repeatedly washing (Note: the outputs were tested by applying a compressive force of 25 N at a contact area of 5x5 cm^2).

Supporting Information 11. Outputs of WPF-MTENG after immersing it in water for several days

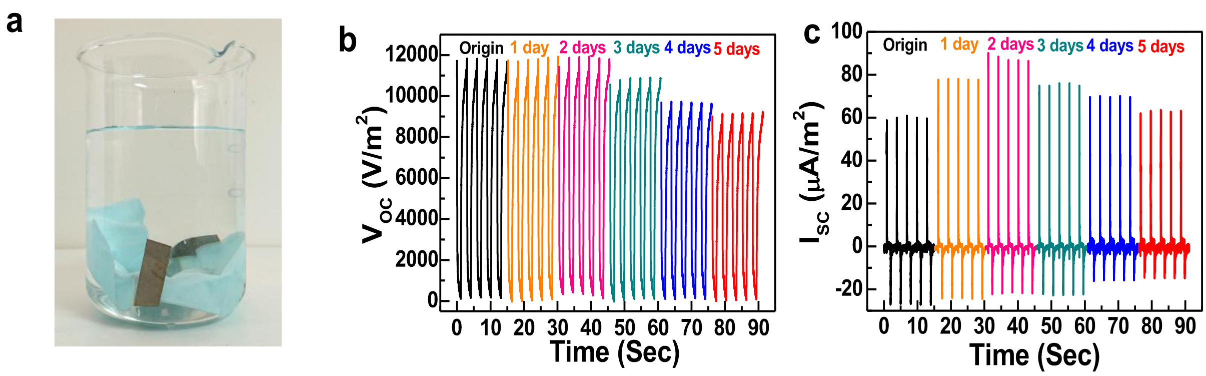

Figure S10. (a) Photo of immersing the WPF-MTENG into water. (b)V_{oc} and (c)I_{sc} of WPF-MTENG after immersing the WPF-MTENG for several days (Note: the outputs were tested by applying a compressive force of 25 N at a contact area of 5x5 cm^2).

Supporting Information 12. I_{sc} of WPF-MTENGs at different structures

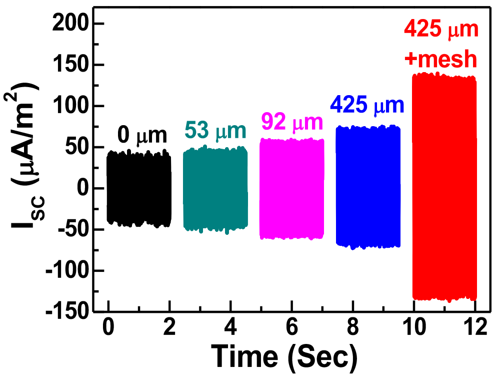

Figure S11. Isc of WPF-MTENGs constructed by rubber membranes molded by different SiC-grit sizes and with/without an additional mesh fabric. Wind speed was set at 14.1 m/s.

Supporting Information 13. Outputs of WPF-MTENG depending on different heights of spacers

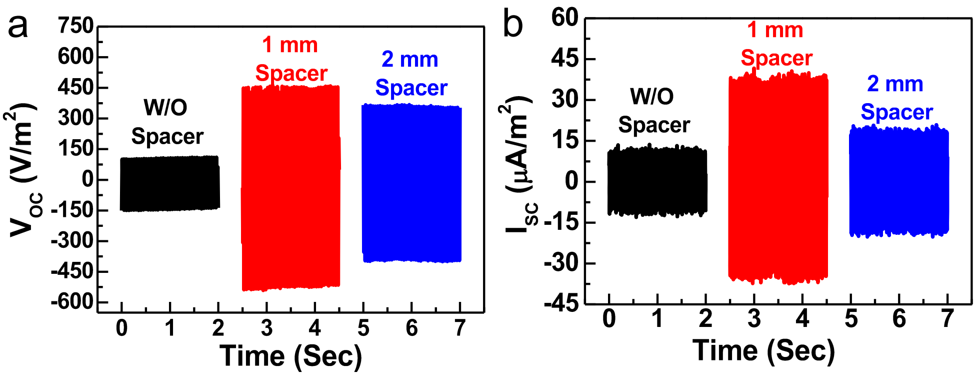

Figure S12. (a) V_{oc} and (b) I_{sc} of WPF-MTENGs with different heights of spacers at 14.1-m/s wind speed.

Supporting Information 14. Output current of WPF-MTENG to different external load

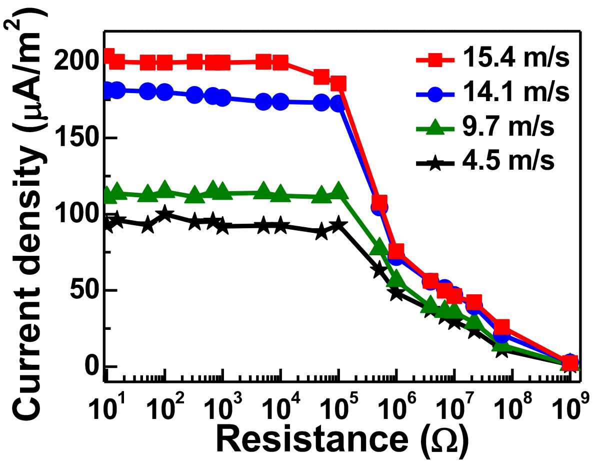

Figure S13. Output current of WPF-MTENG to different external load.

Supporting Information 15. The air permeability of the WPF-MTENG

Figure S14 shows the air permeability of the WPF-MTENG, EVA film, and other commercial breathable and waterproof fabric. Because the outer EVA film is a continuous film, the WPF-MTENG is lack of air permeability. On the other hand, entirely sealing the WPF-MTENG is necessary because the contact and separation of the two active fabrics come from the motion of the air between the fabrics. If the multi-layered WPF-MTENG is breathable, the top fabric will adhere to the rubber membrane after they are in contact, leading to the issue in separating the two active fabrics. However, in practical uses, it is not necessary to fabricate the entire garment by the multi-layered WPF-MTENG. The WPF-MTENG can be joined to other breathable fabrics for designing a power garment. Only the partial regions of a smart garment need to join the fabric-based WPF-MTENG. For example, for the use of body-motion energy harvesting, the WPF-MTENG can be joined on the regions of a garment where the motion of bodies is large, such as the regions on the elbows or knees.

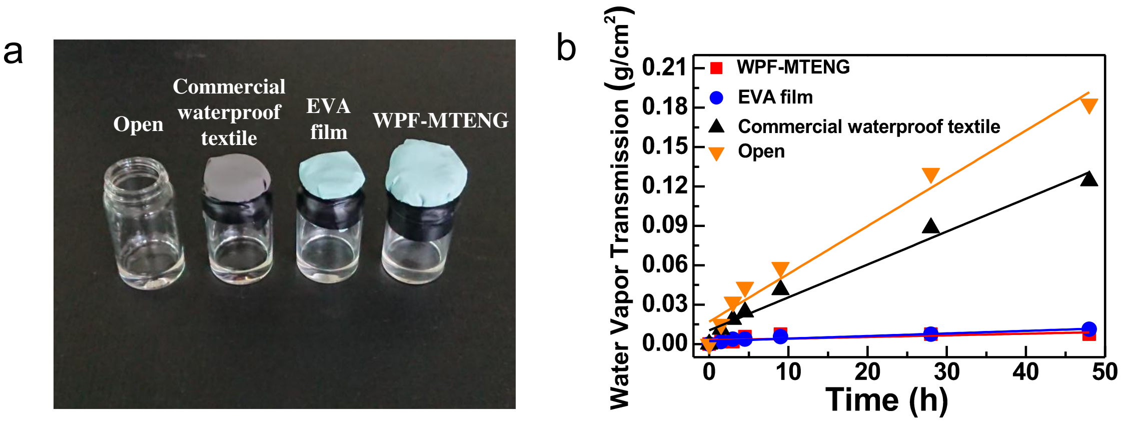

Figure S14. (a) Photo demonstrating of testing the air permeability.(b) Water vapor transmission of different conditions at 24 ℃, RH=54 %.

Supporting Information 16. Durability of WPF-MTENG

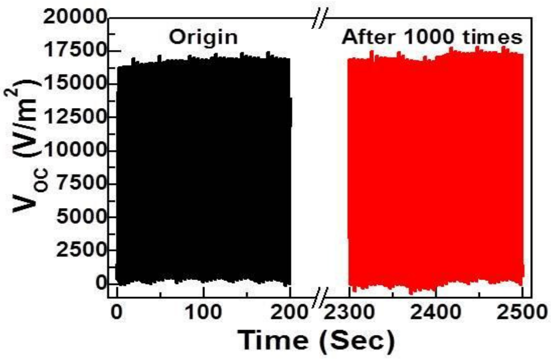

Figure S15 V_{oc} during cyclically loading and unloading a contact force of 30 N (~ 3 kg). The test area is performed under a contact area of 5x5 cm^2.

Supporting Information 17. Circuit diagrams for WPF-MTENG-based self-powered sensors

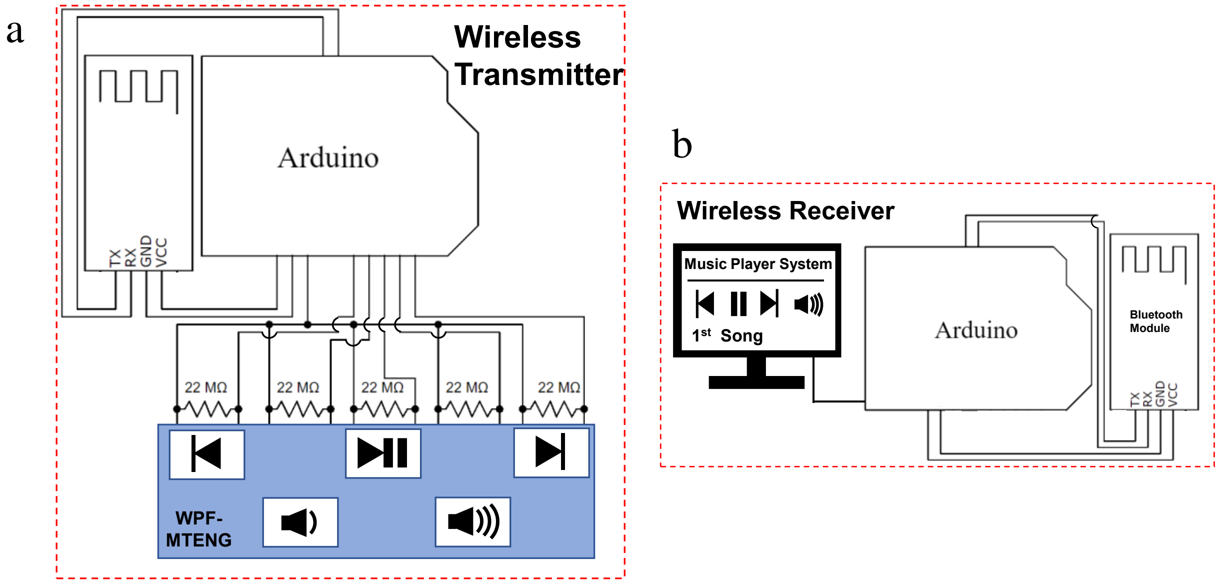

Figure R16 Circuit diagrams for (a) the WPF-MTENG-based self-powered tactile sensors and the wireless transmitter and (b) the wireless receiver.

Supporting Information 18. Demonstrations of WPF-MTENGs for other sensing uses

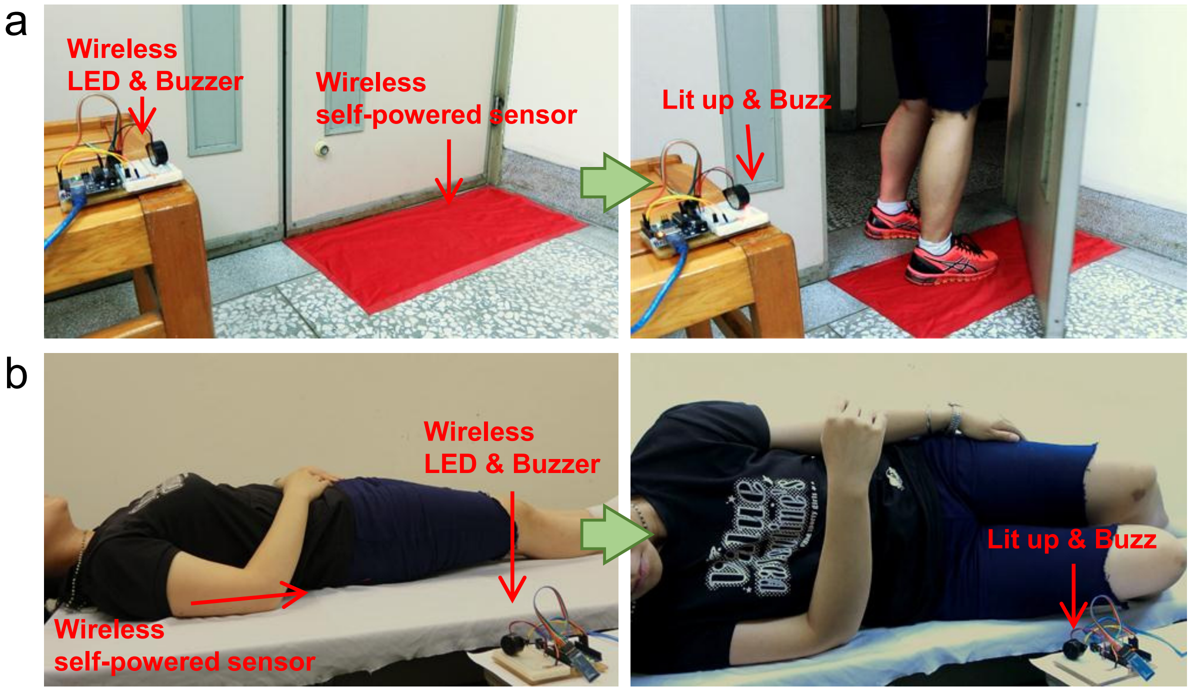

Figure S17. Demonstrations of WPF-MTENGs for the uses of (a) a smart active detecting carpet and (b) an active sensing coverlet.

Reference

[S1] J. Xiong, M. F. Lin, J. Wang, S. L. Gaw, K. Parida, P. S. Lee, Adv. Energy Mater.**2017**, 7, 1701243.

[S2] Y. Liu, N. Sun, J. Liu, Z. Wen, X. Sun, S.-T. Lee, B. Sun, ACS Nano **2018**, 12, 2893.

[S3] Z. H. Lin, G. Cheng, S. Lee, K. C. Pradel, Z. L. Wang, Adv. Mater. **2014**, 26, 4690.

[S4] Z. Zhao, X. Pu, C. Du,; L. Li, C. Jiang, W. Hu, Z. L. Wang, ACS Nano **2016**, 10, 1780.

[S5] J. Bae, J. Lee, S. Kim, J. Ha, B.-S. Lee, Y. Park, C. Choong, J.-B. Kim, Z. L. Wang, H.-Y. Kim, Nat. Commun. **2014,** 5, 4929.

[S6] L. Zhang, B. Zhang, J. Chen, L. Jin, W. Deng, J. Tang, H. Zhang, H. Pan, M. Zhu, W. Yang, Adv. Mater. **2016**, 28, 1650.

[S7] K. Dong, J. Deng, Y. Zi, Y. C. Wang, C. Xu, H. Zou, W. Ding, Y. Dai, B. Gu, B. Sun, Adv. Mater. **2017**, 29, 1702648.

[S8] Y. C. Lai, J. Deng, S. L. Zhang, S. Niu, H. Guo, Z. L. Wang, Adv. Func. Mater. **2017**, 27, 1604462.

[S9] X. Pu, L. Li, H. Song, C. Du, Z. Zhao, C. Jiang, G. Cao, W. Hu, Z. L. Wang, Adv. Mater. **2015,** 27, 2472.

[S10] K. Dong, Y.-C. Wang, J. Deng, Y. Dai, S. L. Zhang, H. Zou, B. Gu, B. Sun, Z. L. Wang, ACS Nano **2017,** 11, 9490.
